# Medical Society of County of New York

**Published:** 1869-10

**Authors:** Geo. T. Elliot

**Affiliations:** President, in the Chair


### MEDICAL SOCIETY OF COUNTY OF NEW YORK.

*Stated Meeting, March 1st, 1869.*

Dr. GEO. T. ELLIOT, Jr., President, in the Chair.

After transaction of the usual preliminary business, and the reading of the reports of committees, a paper was presented by Dr. F. D. Weisse, entitled "Lister's Antiseptic Treatment in Surgery." This paper is published in full in the *Medical Record*, and we therefore present only a summary thereof.

After stating the object of the paper, the author gave an historical sketch of the introduction of the antiseptic treatment of wounds, accrediting Mr. Lister, of Glasgow, with the chief merit of originating and perfecting this system of treatment—although he had been anticipated by many in the use of carbolic acid, for its antiseptic qualities.

The mortality of surgical cases is generally due to one of *four* general causes:—

1. Shock from injury or operation.
2. Consecutive inflammation, with sequelæ of injuries and operations (profuse suppuration, pyæmia, etc.)
3. Heterologous formations (cancer, etc.)
4. Degenerations of tissue which destroy the integrity of organs, such as calcification and atheroma of the coats of arteries, leading to gangrene, aneurism, etc. The second of these general causes is the most prolific source of mortality in surgical practice.

The cause of all these disturbances in surgical cases is usually, in the opinion of Mr. Lister, the irritating and poisoning influence of decomposing blood or sloughs. The essential cause of suppuration in wounds is decomposition, brought about by the influence of the atmosphere upon blood or serum retained within them; and, in the case of contused wounds, upon portions of tissue destroyed by the violence of the injury.

Proof of this is obtained from the fact that, within 24 hours after the accident, the colored serum which oozes from wounds is already distinctly tainted with the odor of decomposition; and during the next two or three days, before suppuration has set in, the smell of the effused fluids becomes more and more offensive.

These putrid fluids may be absorbed, and so give rise to constitutional irritation—the term so generally employed to express the infection of the system from the absorption of poisonous materials.

Microscopical examination of the atmosphere shows conclusively that it contains innumerable germs of living bodies, and the varied experiments, by Pasteur and others, prove that to the presence of these germs in fluids decomposition is due. Prevent these germs absolutely from entering fluids, and no such thing as fermentation or decomposition can take place. Thus the old idea that putrefaction was effected by the oxygen of the atmosphere is exploded.

From this we know that the unhealthiness of crowded hospital wards is due principally to the presence of these germs in the atmosphere.

The claims of antiseptics in surgery are based upon the fact that they have the power of destroying these germs, and thus rendering the air free from septic influence upon wounds.

The power of sulphurous acid in preventing and arresting fermentation and decomposition has long been an established fact. To Polli, of Milan, we owe its therapeutical application. The virtues of choloride of zinc have been advanced by Mr. Campbell de Morgan, of the Middlesex Hospital, London. The sulpho-carbolates of soda, etc., have, of late, been brought forward as more efficient than sulphurous acid preparations, by Mr. Arthur E. Sansom. Carbolic acid outrivals them all, however, as a general local application in surgical practice.

The advantages which Mr. Lister finds that carbolic acid possesses are:

"1. It is a most potent poison to the low forms of life which determine putrefaction, and it retains this power, even though diluted to such a degree as to be almost entirely unirritating to the tissues of the human body.

"2. It is volatile, and its vapor is quite efficacious as an antiseptic upon the air in the vicinity (of wounds).

"3. It is a local anæsthetic, and exercises a most soothing influence upon a painful wound.

"4. It is soluble in a variety of liquids of very different properties—and each of these solutions has its own special value in practice."

The preparations of carbolic acid which have been employed by Mr. Lister have been of various strengths, ranging from the pure acid (liquefied crystals) to a dilution in the proportion of one part of the acid to forty parts of a suitable solvent or vehicle. The glacial or crystalline form is used in all his preparations. (It is solid at ordinary temperatures, but melts readily when heated, or when a little water is added to the crystals.)

In his earlier cases of compound fractures, etc., Mr. Lister used the pure acid (deliquesced crystals); but he renounced it, because it induced too much unnecessary irritation, increasing discharge, and thus retarding cicatrization.

The materials used in the present system of treatment are liquid and solid preparations of carbolic acid, and pieces of block-tin, tin-foil, etc.

Liquid solutions: 1. In water; 2. In oils or glycerin; 3. Alcohol.

Solid preparations: Paste or putty; 2. Plasters; 3. Ligatures.

LIQUID SOLUTIONS.

1. In water: "Water dissolves but a small portion of the acid, only 1-20th part of the pure crystals, and holds that small quantity very loosely, so as to permit it to act with energy on any substance for which it has stronger attractions, and also to become dissipated by exposure." It is applicable as a wash to the interior of recent wounds, whether the result of accident or operation.

2. Oily solutions. (Linseed and olive oil, glycerin.) "The fixed oils have so strong an affinity for the acid, that they will mix in any proportions with it, and hold it so firmly as not to permit it to act with much energy on the tissues, or to become dissipated into the atmosphere."

3. An *alcoholic solution* of one to four has been used. Alcohol readily dissolves the acid crystals, almost equal to the oils: but, like the watery solution, it holds it but loosely. For this latter reason it is applicable to the surface of wounds, etc., especially those that have been exposed for several hours after injury.

SOLID PREPARATIONS.

1. Paste or putty.

This is an oily application made to assume a solid form. Linseed-oil, four parts, carbolic acid (crystals), one part, enough common whitening to form a paste of the consistence of glazier's putty.

2. Plasters.

These are made with olive-oil, litharge, beeswax, and carbolic acid, one to ten, one to twenty, and one to forty. They are reliable, retaining their virtues for many hours. As an external dressing or guard, they shed the discharges freely, and keep all underneath antiseptic. They possess some disadvantages, and have more recently been superseded by what Mr. Lister calls the *antiseptic lac.*

It consists of shell-lac with which carbolic acid will mix in any amount by aid of heat; when mixed in right proportions it may be spread, when cool on calico. In this manner a large quantity of the antiseptic is stored up. It is not softened by the discharges. By brushing over the surface with a weak solution of gutta-percha in bisulphide of carbon, it is rendered non-adhesive. We have in this a durable and perfectly reliable antiseptic external dressing or guard, and at the same time a light and neat one.

3. Antiseptic Ligature.

This is prepared by steeping the silk thread for two hours in strong fluid carbolic acid, prepared by adding a small proportion of water to the crystals. This ligature is applicable to the deligation of main arteries in their continuity, or arteries on the face of a stump or wound.

The antiseptic curtain is a piece of muslin saturated with oily solution of carbolic acid, under which cavities are punctured, to prevent regurgitation of any air except that which has been deprived of its germs by being filtered through a carbolic acid vapor. The antiseptic guard is the external antiseptic dressing, which is a reservoir of the acid, to be replenished from time to time, preventing the introduction of septic germs to the wound, and yet allowing the discharge to flow out under it.

In the manipulations required in carrying out the antiseptic system of treatment, we must ever bear in mind the existence of septic atmospheric germs, and be particularly careful in not allowing the air to have access to the surface of a solution of continuity or cavity before it has been filtered through an antiseptic dressing.

The watery solution should be freely applied to all wounds of whatever character. In compound fractures, care should be taken to have the solution penetrate to all the recesses of the wound; during and after the performance of all surgical operations, the cut surfaces should be washed with it.

The alcoholic solution is to be used in the same cases when some hours have elapsed between the solution of continuity and the application of the antiseptic.

In the performance of operations, all the instruments, probes, knives, etc., should be dipped into a solution one to four of olive-oil before being used. If the operation involves the opening of a joint or serous cavity, drop a one to four solution in the track of your incisions.

In external wounds opening into the pleura or peritonæum, a solution one to four may be introduced by strips of lint into the cavity.

Dr. Weisse closed his paper by a summary of the modifications of our pathological views, which in his estimation were forced upon us as the results of the antiseptic method of treating wounds, and he also gave a *resume* of the various applications of this method.

Dr. E. R. Squibb was called upon by the President, and remarked: I have some knowledge of carbolic acid which I may add to that given in the very interesting paper we have just heard. I confess to a strong prejudice, since first reading Mr. Lister's articles, against what I have regarded as a needless complication of a simple method already well known. Moreover, Mr. Lister's honesty and earnestness grew into fanaticism, and led him to exaggerate and over-dose. At first he was inclined to use the undiluted acid in wounds; but he has now got down to two per cent. in water, and finds this strong enough.

We have been misled a little in the use of the crystallized substance known as carbolic acid. The term has ordinarily been applied to three associated substances, phenyl-alcohol, cresyl-alcohol, and xylic-alcohol, belonging to a series which includes most of the aromatic oils. They have all been pretty well proven to be alcohols, and they are not acids by any definition of this word. Of late, however, it has been disputed by Kekule and others that these substances are alcohols, and this opinion would seem to be gaining ground. Of these three tar-alcohols, the phenyl is crystallizable, and has the lowest boiling point. As these became an object of commercial manufacture, the matter was taken up by a Fellow of the Royal Society, a pupil of Laurence, Mr. F. Crace Calvert. Seeing that there was one of the group which required much chemical knowledge and skill to produce it nicely, and knowing that, should he attempt the manufacture of the others, he would be subject to much competition, he turned all his attention to the production of the most difficult, the crystallized phenyl-alcohol, without knowing that it was the least effective of the three. He had no competition. Up to the time of the investigation of the cattle-plague, the other tar-alcohols were supposed not to be antiseptic at all. When Mr. Crookes, and Dr. Smith, and others, took the matter in hand, they had no desire to damage Mr. Calvert's commercial prospects; but were finally compelled to admit that the phenyl-alcohol had no exclusive virtues. In his first report, Mr. Crookes says that the cresyl-alcohol has been supposed to have no antiseptic properties whatever, but that it seems to be nearly if not quite equal to the crystallized preparation. This remark led me to look into the subject; and some experiments convinced me that the cresyl-alcohol is far more efficient than the phenyl. Mr Parkes says that the impure acid, that is, the whole group together, is better for all purposes to which he has applied it, than are the crystals. Indeed, the whole group as found combined in the old-fashioned creasote, the ordinary creasote of the market, is the thing after all. Its efficiency has never been excelled by any of the separated "acids," and the result of their separation is merely to enhance the cost. As I before remarked, this whole matter of the antiseptic treatment of wounds has been too much complicated. Simple watery solutions would probably produce quite as good an effect as these putties, plasters, lac, etc. With regard to burns, I can claim to speak from experience. A watery solution of creasote, of the strength of about one-half of one per cent., or the creasote-water of the pharmacopœia diluted about one-half, applied on a single thickness of old pocket-handkerchief, will allay the pain of a burn sooner than any thing else. Thus diluted it is a most valuable local anæsthetic. Under this dressing, a burn of the first degree will usually heal without suppuration. One of the second or the third degree will not; nor will it under Mr. Lister's applications. The septic-germ theory of Pasteur, though now commonly accepted in a general way, cannot be made responsible for all the evils of suppuration without grave mistake.

Dr. Jacobi wished to inquire of Dr. Squibb what was the chemical or physical effect of the creasote, undiluted or in strong solution, on healthy tissue?

Dr Squibb: Its prominent characteristic is to coagulate albumen. A strong solution applied to the surface turns the epithelium white, by virtue of this property. The whiteness disappears after a time, and the epithelium becomes again transparent. But this transparency indicates no return to its normal condition. The albumen once coagulated, it is dead, and must peel off. Another curious effect is noticeable: if too strong a solution is applied to a burn, it does not appear to relieve the pain; I have repeatedly seen a one or two per cent. solution fail to give relief. The character of the pain produced by a strong solution is exactly like that of a burn; but in the behavior of the two there is this difference: If the part rendered painful by such an application be held up so as to drain it of blood, the pain is increased till it may become almost insupportable; if held down so as to allow the influx of blood, the pain is diminished; herein the pain is like that from chloroform, and is exactly opposed to that from a burn.

I might add that the reason why anatomical specimens are so often rendered opaque and unfit for microscopic examination, by being preserved in carbolic acid, is doubtless the too great strength of the solution employed. I suppose an aqueous solution of one-fourth of one per cent. of the cheap impure acid ought not to injure a delicate specimen. I have now in progress a series of experiments to determine this point, using solutions varying from one-tenth of one to one per cent., which last, I am convinced, is quite too strong. The best test of the proper strength of your solution is its application to the tongue. If it coagulates the albumen, producing an effect like that of hot tea or coffee, it is too strong.

Dr. Jacobi: I am glad to see, Mr. President, that the chemist agrees perfectly with what many of us have repeatedly seen. I have made the same observation with regard to the pain, and the effect of position upon it. I have felt, too, that, if the antiseptic treatment is to be of any use, it must be simpler than Mr. Lister has made it, for by his plan each patient requires five or ten times as much attendance as usual. I have myself used carbolic acid in a great number of instances, both upon living and upon dead tissue. A solution of one-tenth applied to croupous membrane will cause it to shrivel up in a very short time; and the very beneficial effect of the acid upon diphtheritic membranes is due to its coagulating power. But this very property leads me to think that solutions of one-fifth to one-twentieth applied to wounds, etc., as in Mr. Lister's practice, will have an effect just the reverse of what he intends. He wishes to preserve healthy tissue; I think I have often destroyed it by using a solution too strong. I know that a number of years ago, when I knew less about the subject than I do now, I very speedily destroyed the cornea in a case of diphtheritic conjunctivitis. I applied a very thin layer of a dilution of one part to eight or ten of glycerine and water to the conjunctiva; the cornea thus became slightly touched by it, and the result was that it was perforated much earlier than in the normal course of the disease. The diphtheritic affection might have caused perforation in thirty-six hours, while I had done it in five or six. I have seen the shrinking and destruction of healthy tissue immediately follow the use of strong solutions; and I agree with Dr. Squibb, that we have not yet reached the minimum of strength desirable. I now commonly employ a dilution of three or four grains to the ounce. I have used it to wash out the uterus in puerperal endometritis; and I am confident that in many cases of this kind, which have lately come under my hands, the patients have owed their lives to this treatment. In very bad cases, I have used intra-uterine injections of twenty grains to the ounce; but then I am careful to use very little of the injection, to throw it directly into the uterus, and immediately to wash out the vagina with a milder solution, to avoid the unpleasant effect which the stronger one would produce upon it. In cases of common catarrh of the external ear, which are often as obstinate as they are uncomfortable, I have employed a three or four-grain solution, and found them yield more speedily than to any thing else.

Dr. Chadsey had made use of carbolic acid from its first introduction, always in weak solutions, never stronger than one part to forty. A severe burn of the second degree had healed without suppuration under a dressing of the acid covered with oiled silk. He had gained happy results in gunshot wounds, putrid sores, disease of the ear, leucorrhœa, and gonorrhœa. For the latter cases, glycerine rather than oil should be used as a solvent, to avoid staining the linen.

Dr. Smith wished to know whether tissue, whose albumen has been coagulated by any means whatever, could possibly retain its vitality; and if not, how such devitalized tissue was gotten rid in wounds subjected to Lister's treatment?

Dr. Weisse thought it was carried away by absorption, and that the result s proved this.

Dr. Jacobi doubted whether albumen so coagulated would find any solvent in the fluids of the wound; and if not, then its absorption was impossible, for absorption could take place only after solution or fatty degeneration.

Dr. Griscom had given inhalations of carbolic-acid vapor in a case of abscess of the lung, with a happy effect.

Dr. Weisse, referring to the statement that Mr. Lister had come down from the use of the pure alcohol to that of a two-per-cent. solution, said that he would doubtles come lower yet, only he wished to feel his way carefully, without incurring in any case what he woud deem a risk to the patient. Mr. Lister by no means claimed that all suppuration was due to septic germs. He simply said that experience had taught him that you will limit suppuration in a wound if you prevent decomposition of its fluids.

Dr. Stein had made considerable use of the agent. He preferred tin-foil to protect the dressings, as it could be most nicely adapted to the surface. He had assisted in the removal of nearly the entire frontal bone, for necrosis supposed to be syphilitic, the soft parts having previously sloughed. The suppuration, which was excessive, was checked under the application of carbolic acid, and the wound healed very well. Iodide of potassium was freely given.

Dr. Chamberlain, attempting on one occasion to use what he believed to be the impure acid, had found it quite unmanageable, of about the thickness and color of dark molasses, nearly insoluble in water, and indelibly staining the vessel. He wished to know if he had obtained the right article.

Dr. Squibb: "That, sir, is not the 'impure' but the 'crude' carbolic acid. Nothing should ever be used for medical or surgical purposes which is not transparent, though it may be dark. The impure acid is made by redistilling the crude; it is a combination of the three alcohols, and would much better be called creasote, for it is identical with the old-fashioned coal-tar creasote. A mistake often made is that of prescribing a solution, and getting this; and fearful accidents have occurred from its application pure to burns, etc. The safest mode is to dispense the crystals by measure. The impure acid is never wholly soluble in water, but it should leave merely a slight scum. The tar and oils are rendered more soluble by the addition of alkalies, an occasional adulteration."

"Having answered Dr. Chamberlain, I would say that Mr. Lister began at the wrong end, with the heroic style of treatment. I think it a very interesting question—what did become of the coagulated albumen sealed up by him in his early operations; for he used a large amount of the acid, dipped his finger into it, and smeared it all around inside the wound. If we could suppose pepsine to be introduced there, and a process of digestion to be set up, one might look for absorption. I think it most probable that the coagulated material, in such cases, becomes encysted, like other foreign bodies, for it is, to all intents, a foreign body. Mr. Lister's system illustrates what seems to be a strong tendency in human nature—to seek complication. I am constantly finding persons leave the simple solution I have recommended for burns, and go to ointments, etc., which are not only useless but hurtful."

THE PRESIDENT: "I may say that, two years ago last October, I commenced to direct that the wood-work of all the lying-in wards of Bellevue Hospital should be daily dampened with a solution of the crystals, two grains to the ounce. I afterward replaced this by a cheaper preparation of about the same strength. We have since a great diminution in the mortality there; but I would not ascribe this to carbolic acid alone, for we are very careful in our hygiene. In addition, I always direct that every woman in the lying-in ward shall have her vagina thoroughly disinfected. I make it the duty of the interne to see that no woman has any smell of the lochia about her; and for that purpose we have used carbolic acid, Labarraque's solution, and the permanganate of potassa. The last I have now discarded, because the specimens furnished were found irritating, perhaps from some admixture of caustic potash. I am not prepared to say that the carbolic acid is preferable to the Labarraque. Moreover, whenever a woman's vagina is found difficult to disinfect—and we meet with such cases now and then—it is made the nurse's duty to have a large piece of rag, saturated in a solution of the acid, laid near the vagina.

We do not rely upon these means alone; when the floors are washed, a bottle of Labarraque's solution is added to the bucket of water; pans of chloride of lime are placed in the wards, and also shallow dishes of carbolic acid; the windows are kept open day and night, and the doors nearly all the time. It is certain that, by one or all of these means, we are getting clear of puerperal fever in that hospital.

"As to the manner in which carbolic acid checks suppuration, it at this moment occurs to me, as a matter worth investigating, whether it may not possibly so affect the capillaries of the part to which it is applied, as to prevent the lymph globules from escaping."

The Society then adjourned.

—*New York Medical Journal.*

